# Conventional and Non-Conventional Roles of Non-Muscle Myosin II-Actin in Neuronal Development and Degeneration

**DOI:** 10.3390/cells9091926

**Published:** 2020-08-19

**Authors:** Míriam Javier-Torrent, Carlos A. Saura

**Affiliations:** Institut de Neurociències, Department de Bioquímica i Biologia Molecular, Centro de Investigación Biomédica en Red Enfermedades Neurodegenerativas (CIBERNED), Universitat Autònoma de Barcelona, 08193 Barcelona, Spain; miriam.jtorrent@gmail.com

**Keywords:** myosin, actin, Rho GTPase, cytoskeletal motors, actomyosin, nucleokinesis, synapse, neuronal polarization, synaptic plasticity, brain injury, neurodegeneration, intellectual disability

## Abstract

Myosins are motor proteins that use chemical energy to produce mechanical forces driving actin cytoskeletal dynamics. In the brain, the conventional non-muscle myosin II (NMII) regulates actin filament cytoskeletal assembly and contractile forces during structural remodeling of axons and dendrites, contributing to morphology, polarization, and migration of neurons during brain development. NMII isoforms also participate in neurotransmission and synaptic plasticity by driving actin cytoskeletal dynamics during synaptic vesicle release and retrieval, and formation, maturation, and remodeling of dendritic spines. NMIIs are expressed differentially in cerebral non-neuronal cells, such as microglia, astrocytes, and endothelial cells, wherein they play key functions in inflammation, myelination, and repair. Besides major efforts to understand the physiological functions and regulatory mechanisms of NMIIs in the nervous system, their contributions to brain pathologies are still largely unclear. Nonetheless, genetic mutations or deregulation of NMII and its regulatory effectors are linked to autism, schizophrenia, intellectual disability, and neurodegeneration, indicating non-conventional roles of NMIIs in cellular mechanisms underlying neurodevelopmental and neurodegenerative disorders. Here, we summarize the emerging biological roles of NMIIs in the brain, and discuss how actomyosin signaling contributes to dysfunction of neurons and glial cells in the context of neurological disorders. This knowledge is relevant for a deep understanding of NMIIs on the pathogenesis and therapeutics of neuropsychiatric and neurodegenerative diseases.

## 1. Introduction

Myosins are motor proteins that produce tension and contractile forces for movement and sliding of actin filaments in the majority of cells of human tissues. By affecting the dynamics of the cell’s cytoskeleton, the actin/myosin complex (actomyosin) contributes to cell division, adhesion, polarity, contractility, migration, endocytosis, and vesicular transport. The superfamily of myosin proteins contains up to 40 genes in mammals, including conventional and non-conventional myosins [1]. It is then not surprising that myosins, and in particular the conventional non-muscle myosin II (NMII), participate in multiple tissue functions and in human diseases ranging from cancer to neurological disorders [2,3,4,5].

**Figure 1:**
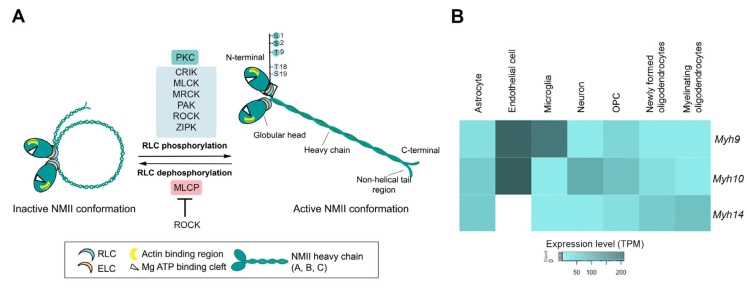
Structure, regulation, and cellular expression of non-muscle myosin IIs (NMIIs). (**A**) Schematic structure of NMII and its regulation by phosphorylation. The NMII holoenzyme consists of a globular head motor domain at the N-terminal, containing the actin (yellow) and Mg^2+^-ATP (white) binding sites, followed by an isoleucine-glutamine (IQ) neck region bound to the essential light chains (ELC) and regulatory light chains (RLC), and coiled-coil tail region consisting of a heavy chain (HC) that ends with a non-helical tail region at the C-terminal that defines the subcellular localization. Two NMII holoenzymes form a homodimer by binding via the α-helical coiled-coil tail regions. NMII transits between inactive (left) and active (right) conformations regulated by phosphorylation/dephosphorylation of RLC by several protein kinases (highlighted in green) and myosin light chain phosphatase (MLCP). Phosphorylation of several Ser (S) and Thr (T) sites in RLC regulates the enzymatic activity of myosin. CRIK: citron Rho-interacting kinase; MLCK: myosin light chain kinase; MRCK: myotonic dystrophy-related Cdc42-binding protein kinase; PAK: p-21 activated kinase; PKC: protein kinase C; ROCK: Rho-associated protein kinase; ZIPK: leucine zipper interacting kinase. (**B**) Relative expressions of *Myh9*, *Myh10,* and *Myh14* in the mouse’s cerebral cortex. Expression of NMII isoforms in cells of the cerebral cortex analyzed by single-cell transcriptomic analyses. OPC: oligodendrocyte progenitor cells; TPM: transcripts per kilobase million. Data obtained from the open science resource *Expression Atlas* (https://www.ebi.ac.uk/gxa/).

The NMII holoenzyme consists of a globular head motor domain at the N-terminal, containing the actin binding and Mg^2+^-ATPase sites, followed by a neck domain bound to the essential light chain (ELC) and regulatory light chain (RLC), a tail region consisting of an α-helical heavy chain (HC), and a non-helical tail at the most C-terminal end (Figure 1) [2,3]. The α-helical coiled-coil domain forms homodimers that assemble with a high diversity of myosin light chains, forming filamentous structures with F-actin [6]. In vertebrates, the NMII isoforms differ in their corresponding HCs, which are encoded by three distinct genes: *MYH9* (NMHC IIA; NMIIA)*, MYH10* (NMHC IIB; NMIIB), and *MYH14* (NMHC IIC; NMIIC). NMII self-assembles into bipolar thick filaments that bind to F-actin to mediate motor activity through ATP hydrolysis. The assembly, activity, and motor function of NMII are tightly modulated by dynamic assembly/disassembly transitions regulated by distinct post-translational mechanisms, including phosphorylation of RLC and HC domains, and protein interactions, as described in recent reviews [3,4,7] (Figure 1). Briefly, the assembly-incompetent NMII form is unfolded via phosphorylation of RLC at Thr 18 and Ser 19 by two main kinases: myosin light chain kinase (MLCK) and Rho-associated protein kinase (ROCK) (Figure 1A). Additionally, other kinases mediate RLC phosphorylation at Thr 18 and/or Ser 19, including citron Rho-interacting kinase (CRIK), death-associated protein kinase (DAPK3), myotonic dystrophy-related Cdc42-binding protein kinase (MRCK), serine/threonine-protein kinase 21 (STK21), p-21 activated kinase (PAK), and leucine zipper interacting kinase (ZIPK). In contrast to these activating phosphorylation sites, protein kinase C (PKC)-mediated RLC phosphorylation at Ser 1, Ser 2, and Thr 9 results in decreased myosin activity. The HC is also phosphorylated by multiple kinases, including PKC, casein kinase II (CKII), and transient receptor potential melastatin-7 (TRPM7). PKC-mediated phosphorylation of the HC on Ser 1916 and Ser 1937 and CKII on Ser 1943 prevent assembly of myosin into filaments. Distinct signaling pathways regulate these phosphorylation sites, but how these modifications are integrated to induce physiological responses by particular NMII isoforms in neurons and glial cells has yet to be elucidated and requires further investigation.

The diversity of NMII subtypes and regulatory mechanisms, and their differential expression in distinct cell types. increase their functional complexity in the nervous system [8]. The generation of contractile forces mediated by the actomyosin filaments in neurons directly regulates the morphology and growth of axons and dendrites [9], and synapse morphology and plasticity [8]. Besides their classical functions in neurons, NMIIs play important roles in glial cell biology processes, such as inflammation, myelination, repair, and blood–brain barrier integrity. Of relevance, beyond the non-conventional roles in cellular processes severely affected in neurodevelopmental and neurodegenerative disorders, genetic mutations in NMIIs and signaling factors regulating their activity have been recently linked to neuropsychiatric diseases, such as autism spectrum disorders, schizophrenia, and intellectual disability (recently reviewed in [5]).

This review describes the emerging roles of actomyosin cytoskeleton in brain development and degeneration, focusing mainly in NMII biology and regulatory molecular mechanisms in neurons and glial cells. We discuss the function of actomyosin signaling in neuronal morphology, polarization, migration, and plasticity, and its involvement in neuron dysfunction and death in the context of cognitive and behavioral alterations in neuropsychiatric and neurodegenerative diseases.

## 2. Non-Muscle Myosin II in the Nervous System

### The Cerebral Expression and Cellular Localization of NMIIs

NMII isoforms are differentially expressed in human tissues, including the central nervous system (CNS) [10,11,12,13,14] (summarized in Table 1). Notably, alternative splice variants of *MYH9* and *MYH10*, containing exon insertions in the head domain near the ATP motor domain (loop 1) and actin-binding region (loop 2), are expressed selectively in the nervous system [12]. The *MYH10 loop 1* variant is abundant in the fetal brain, whereas the *loop 2* variant appears just after birth and is strongly expressed in the adult brain [15]. By contrast, the *MYH14 loop 1* variant is abundant in the liver, although it is present in other tissues, including the brain [13]. Despite their differential expression during brain development and adulthood, it is not clear whether these splice variants are cell-type specific and have specific functions in the developing and adult CNSs.

Proteomic analysis of the mouse’s cerebral cortex shows that the relative abundances of NMIIA, B, and C are 29%, 67%, and 4% [14]. This differential expression of NMIIs may reflect the cell complexity of the nervous system and specific roles of NMIIs in distinct cerebral cells and regions. The initial biochemical analyses did not provide information about the cell-type specific expression of NMII isoforms in the brain. State-of-the art single-cell transcriptomic analysis of the murine cerebral cortex has recently revealed that *Myh9*, *Myh10*, and *Myh14* are the most abundant isoforms in microglia, neurons, and oligodendrocytes, respectively (Figure 1B) [16]. All three isoforms are similarly expressed in astrocytes, whereas *Myh9* and *Myh10* are expressed also in endothelial cells, where they regulate brain vasculature [17]. These results agree with the high abundance of NMIIB in neurons and glial cells in the rat, mouse, and bovine brains [18]. This could explain why NMIIB plays a prominent role in neuronal morphology, migration, and plasticity (see below). NMIIB is not restricted to neurons in the adult brain. NMIIB is not highly expressed in microglia but it plays a pivotal role in cytoskeletal organization during microglial activation [19]. On the other hand, NMIIB is highly expressed in oligodendrocyte precursors and then declines during differentiation, which suggests that NMIIB participates in maturation and myelination of oligodendrocytes. In support of this idea, genetic or pharmacological NMIIB inactivation accelerates maturation, branching, and myelination of oligodendrocytes [20,21]. It is interesting to point out that *Myh14* is the less abundant isoform in oligodendrocyte precursors but its expression increases in new and mature oligodendrocytes, which suggests a role of this isoform in myelination.

**Figure 2:**
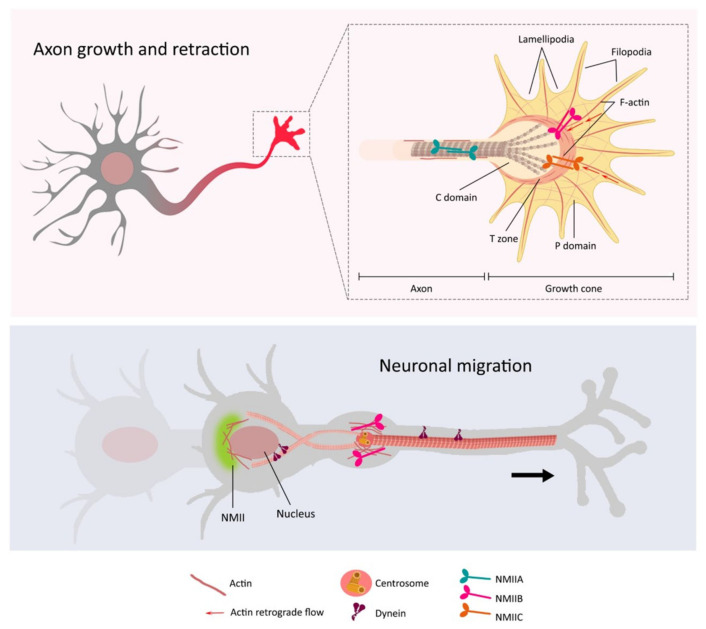
NMIIs regulate axon dynamics and neuronal migration. Actomyosin activity is critical for cytoskeletal dynamics during axon outgrowth/retraction and neuronal migration. The images depict the localization of NMII isoforms in different neuronal compartments, including the axon and growth cone (top), and leading process and soma (bottom). **Top**: A neuron extends and retracts the axon by means of cytoskeletal modifications mediated by NMII/actin motors at the growth cone (in red; magnified it at the right); actin filaments are assembled and retrogradely transported by NMII at the P domain, allowing extensions of the filopodia and lamellipodia; actin filaments are recycled and disassembled at the T zone; and NMII/F-actin guide microtubules at the C domain to initiate a forward movement towards the growing site. **Bottom**: NMII mediates actin filaments dynamics to provide the pulling forces to translocate the soma and the nucleus during neuronal migration. NMIIB and actin are enriched in the neuronal leading process surrounding the centrosome and the trailing process, where they coordinate the forward movement of the centrosome and soma in the direction of the migration. NMII isoforms accumulate at the rear part of the soma, where they are assisted by dynein to push the nucleus forward along the trailing microtubules in migrating neurons.

How NMII isoforms regulate actin dynamics in specific subcellular regions to facilitate cell morphological and functional changes is an important unanswered question. This issue is particularly important in polarized cells, such as neurons, which require cellular compartmentalization to ensure propagation of chemical and electrical signals along neural circuits. The subcellular location of NMIIs is determined by the C-terminal non-helical region of the HC [22,23], and dictates the different rates of assembly and disassembly of each isoform [24]. Neurons receive synaptic inputs on the soma and dendrites, and then the action potential generated at the axon initial segment (AIS) propagates along the axon towards the synapse terminal and to downstream neurons (Figure 2). In developing neurons, microtubule and actomyosin-mediated morphological changes at the end of the distal axon (i.e., growth cone) mediate axon growth and retraction. Initial seminal studies demonstrated high levels of NMIIA and NMIIB at the soma and the actin-rich growth cones of dorsal root ganglion (DRG) neurons [25,26,27]. Although NMIIA and NMIIB evenly overlap at growth cones, the latter is usually more abundant at the transition (T) and periphery (P) regions, where myosin highly colocalizes with actin filaments [27,28] (Figure 2). This differential localization may explain the opposite roles of NMIIB and NMIIA in retrograde actin flow in the growth cone during axon guidance and growth [29,30] (see also below). Remarkably, NMIIA/B distribution at the growth cone is disrupted by blebbistatin [28,29], an inhibitor of muscle and non-muscle myosin II that stabilizes the myosin ADP·Pi complex interfering with ATP hydrolysis and cycle completion [31,32]. These results provide a structural basis for the effects of blebbistatin on axon guidance and elongation. Specific localization of NMIIs at the growth cone may therefore be critical for growth cone dynamics and axon elongation during neuronal development.

NMIIs regulate the migration of developing neurons, and the morphology and function of mature neurons. During neuronal migration, F-actin and NMIIB are enriched at the proximal region of the neuron’s leading process in the direction of migration, where they regulate the force generation that drives soma and nuclear translocation [33,34] (Figure 2). Particularly, active phosphorylated RLC is concentrated at the soma and the distal regions of the leading and trailing processes, which are the main contraction centers participating in the traction forces during migration of granule cerebellar neurons [35]. Interestingly, NMII localization changes during the maturation of neurons. This is exemplified by the localization of NMIIB at the soma and proximal branch points of dendrites in immature hippocampal neurons, and its redistribution to the neck and head of dendritic spines in mature stages [36,37]. Despite the fact that NMIIB is the most abundant isoform in neurons, all three NMIIs are expressed differentially in axonal and somatodendritic compartments in cultured hippocampal neurons [38]. These changes in the subcellular localization of NMII isoforms during neuronal differentiation and maturation likely reflect their distinct compartmentalization and functions in neurons.

NMII is also an integral component of the AIS, a macromolecular complex region that connects the somatodendritic compartment with the axon that contributes to the generation of action potentials, electrical excitability, and axodendritic polarity [39]. During the maturation of hippocampal neurons, phosphorylated RLC decreases activity in the axon and increases it in the AIS, where it associates with F-actin [38]. Indeed, RLC phosphorylation and myosin contractile activity are required for AIS complex assembly and stability, and the activity-dependent relocation of AIS components along the axon, indicating a role for NMII activity in AIS plasticity [38,40]. The specific NMII isoforms and regulatory mechanisms that mediate AIS structure and function have not been identified. It is possible that NMIIs contribute to neuron polarity and morphology not only by acting on the distal axon and growth cone but also regulating the formation and organization of the AIS. Considering that AIS is affected in several neurological disorders [41], the contribution of NMII to AIS dysfunction represents an important area of investigation.

## 3. Functions of Non-Muscle Myosin II in the Nervous System

### 3.1. Non-Muscle Myosin II in Neuronal Polarization

#### 3.1.1. The Role of NMII During Brain Development

Studies in mice deficient in *Myh9*, *Myh10,* and *Myh14* indicate a critical role for specific NMIIs in brain development. Germline *Myh9* deletion is embryonically lethal due to adhesion defects and the lack of maturation of the visceral endoderm [42]. This phenotype is rescued by expressing *Myh10*, but the resultant mice die later at embryonic day 9.5 due to defects in angiogenesis and cell migration that lead to abnormal placenta formation [43]. By contrast, *Myh10* knockout mice develop severe cardiac failure and brain abnormalities, including hydrocephalus and defects in cerebral neuroepithelium and cell migration, which result in late embryonic lethality [44,45,46]. Interestingly, this brain phenotype, but not the neuronal migration defects, is rescued by expressing *Myh9* or the enzyme activity-deficient NMII R709C mutant [45,47,48,49]. This suggests that structural rather than enzymatic motor activity maintains cell-cell adhesion during brain development, whereas certain functions, such as neuronal migration, require NMIIB function. Indeed, mutant mice harboring a point mutation in the motor domain of *Myh10* develop locomotor impairments associated with disrupted migration of cerebellar granule cells [50]. These results indicate that NMII-mediated neuronal migration in the cerebellum during development is essential for motor function, but whether mutant *Myh10* causes physiological defects in other brain regions is still unclear.

The generation of cell-specific knockout mice has recently provided new insights into certain NMIIs physiological functions. Mice lacking *Myh10* conditionally in neurons show postnatal lethality without cardiac defects, whereas brain abnormalities are not observed in mice lacking *Myh10* in myocytes [51]. This suggests overlapping and partial compensatory functions of the most abundant isoforms, *Myh9* and *Myh10*, in the heart and brain during embryonic development. In contrast to *Myh9* and *Myh10* knockout mice, *Myh14* deletion does not result in embryonic lethality or brain and cardiac defects [14]. This indicates either a minor function of *Myh14* during brain development and/or compensatory function by the other isoforms. Whether *Myh14* plays particular roles in specific cell types of the CNS is still unclear. For instance, *Myh14* levels increase during oligodendrocyte differentiation, which suggests a role for this isoform in myelination. This may explain the requirement of NMII activity for the branching and myelination of oligodendrocytes, and segregation and myelination of axons by Schwann cells [21]. Considering the differential expression of the three NMII isoforms in the brain, it is critical to determine their specific contributions to neuronal and glial physiology. Generation and characterization of cell-type specific NMII knockout mice will be useful for deciphering the physiological functions of NMII isoforms in the nervous system.

#### 3.1.2. The Function of NMII in Neuronal Polarization

Neuronal polarization requires constant changes in the dynamics of the cytoskeleton at the distal region of the neurite, including the growth cone. This region undergoes constant extension and retraction processes that rely on local NMII activity [52,53], and determine neuronal polarity and axon outgrowth [54]. The distal part of the growth cone is divided into three regions: (1) the peripheral domain (P domain) that contains the transient structures filopodium and lamellipodium, where actin filaments are assembled and retrogradely transported by NMII; (2) the transition zone (T zone), where actin filaments are recycled and disassembled; and (3) the central domain (C domain) that is composed of microtubules and contains organelles and vesicles (Figure 2). Axon growth comprises three main cycle stages occurring at the growth cone: protrusion, engorgement, and consolidation, and all three steps require the involvement of NMII activity (reviewed in [55]) (Figure 2). Briefly, during protrusion, the actin retrograde flow, which is highly dependent on NMII, is reduced, allowing filopodia and lamellipodia to move forward to the P domain [29,56]. As engorgement occurs, transverse F-actin arcs guide microtubules to initiate a forward movement towards the growing site. Finally, during consolidation NMII-containing actin arcs compress microtubules in the C domain and cease the F-actin protrusive activity, leading to the retraction of filopodia and the elongation of the axon shaft [30].

**Figure 3:**
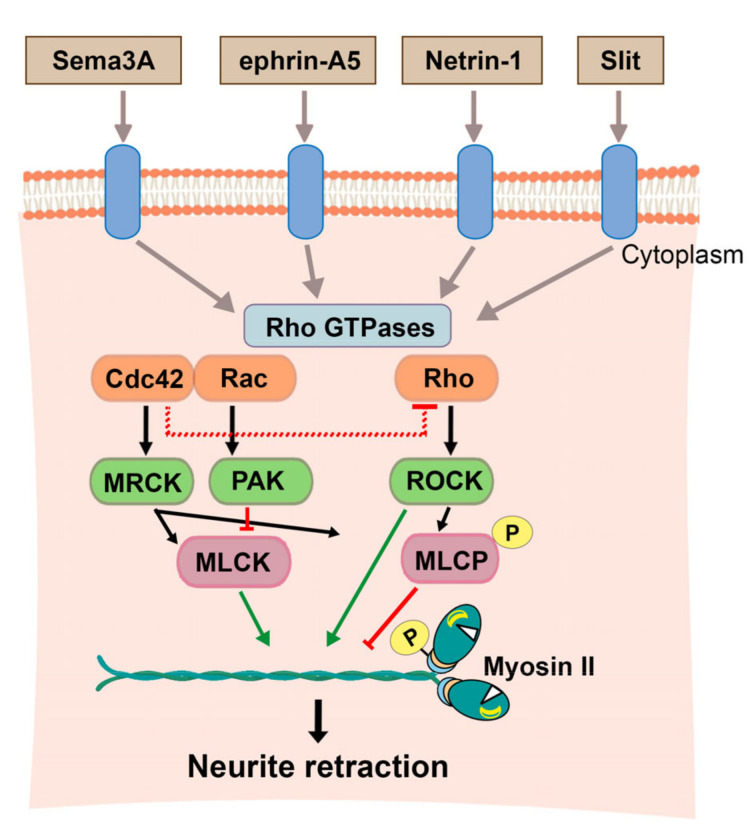
Signaling pathways leading to NMII-mediated regulation of neurite outgrowth. Several guidance repulsive cues, including Sema3A, ephrin-A5, Netrin-1 and Slit, activate RhoGTPases Cdc42, Rac and RhoA to regulate kinases or phosphatases affecting NMII phosphorylation and contractility. **Right**: RhoA activates its effector ROCK, which phosphorylates directly NMII. ROCK also inactivates myosin light chain phosphatase (MLCP) resulting in actomyosin contractility, growth cone collapse and neurite retraction. **Left**: Cdc42 activates MRCK leading to MLCK-mediated NMII phosphorylation and neurite retraction. Rac activates PAK that phosphorylates and inhibits MLCK to mediate neurite extension. Black/green arrows: activation; Red arrows: inhibition.

Several signaling pathways regulate neuronal morphology, synaptogenesis, and axon growth and guidance by converging on the Rho GTPases (Figure 3). The family of Rho GTPases work as signaling bridges that link cell surface receptors to the organization and structural changes of actin and microtubule cytoskeletons in neurons [57]. NMII, acting as a downstream target of Rho GTPases, plays a central role in cytoskeletal dynamics in these processes. For instance, MLCK and RhoA-mediated NMII phosphorylation and activation negatively regulate the polarity of hippocampal neurons, whereas NMII inhibition accelerates neuronal polarity [53]. Some studies show that NMII activation through G-protein coupled receptors regulates somatodendritic morphology by mediating actomyosin contraction. Activation of RhoA mediates growth cone collapse and neurite retraction in response to repulsive signals. Similarly, acute activation of CB1 cannabinoid receptor (CB1R) induces rapid RhoA/ROCK-mediated retraction of distal and proximal dendrites in hippocampal neurons [58]. In summary, Rho GTPAses regulate cytoskeletal dynamics by affecting, among others, actomyosin activity during neuronal polarity in developing neurons.

Which are the roles of NMIIs in axon growth? Genetic and pharmacological studies have shown a critical role, and sometimes even opposite functions, of NMII isoforms in neuronal adhesion and growth cone dynamics [28,29,59,60,61,62,63] (Figure 2). NMIIA is a downstream effector of the Rho GTPase signaling pathway during neurite retraction [64]. Disruption of NMIIA activity induces a rearrangement of the actin cytoskeleton and loss of focal contacts that leads to an increase of axon length in DRG neurons [28,63,65,66], whereas overexpression of a non-phosphorylable NMIIA mutant that promotes myosin assembly impairs axon elongation in hippocampal neurons [59]. By contrast, NMIIB facilitates neurite and axon growth, as revealed by decreased neurite outgrowth by NMIIB knockdown in neuroblastoma cells [63,65], and impaired axonal outgrowth and growth cone morphology in *Myh10* knockout mice [55]. Similar to NMIIA, NMIIC modulates cell adhesion in neuronal cells, but knockdown experiments demonstrate that NMIIC drives neurite outgrowth as NMIIB does [62]. Together, these results indicate both distinct and overlapping roles of NMII isoforms in the regulation of axon growth.

Several observations suggest different non-exclusive possibilities of how the three NMII isoforms interplay during axon elongation and retraction process. First, NMII isoforms localize differentially at the growth cone: NMIIA is enriched in the central domain, and NMIIB and NMIIC are localized in the transition zone where kinesin-5 opposes dynein-driven forces on microtubules [67]. Notably during collapse, RhoA activity determines the localization of each isoform within the growth cone. Thus, in response to semaphorin 3A (Sema3A), NMIIA redistributes from the growth cone to the neurite of DRG neurons, causing actin instability, which is followed by NMIIB relocalization at the rear of the growth cone, where it associates with actin to drive axon retraction [68]. Another possibility is that when Rho activity is low, the preference to activate NMIIA switches to NMIIB or NMIIC, as observed in other cell types [69]. Since many guidance cues mediate axon retraction in different neuronal subtypes, it is possible that they may modulate the strength of the response that leads to the activation of Rho signaling that in turn modulates NMII activity (Figure 3). Some evidence that supports this model includes: (i) slit and netrin-1 mediate growth cone collapse in cranial motor neurons [70], (ii) ephrin-A5 induces growth cone collapse and axon retraction in hippocampal and retinal ganglion neurons [59,71,72]; (iii) the nerve growth factor (NGF) controls growth cone polarity and guidance in forebrain neurons [53]; and (iv) all these functions rely on the activation of RhoA and MLCK signaling. These studies also support the idea that guidance cues may contribute differentially to neuronal polarization depending on the neuronal type. The emergence of transcriptomics analyses opens up the path to investigate whether the activation of these pathways depends on the trophic factors or guidance cues gradients, or whether the response of each neuronal type is established by specific cues.

On the other hand, NMII activity affects axon growth and guidance, depending on the cellular stimuli and context. For instance, the axon growth-promoting effect of blebbistatin depends on NMIIA in permissive substrates, whereas it is mediated by both NMIIA and NMIIB in inhibitory substrates [28]. Rho signaling plays a central role in mechanotransduction, a process that converts mechanical stimuli to biochemical responses. While neurite growth of hippocampal neurons is dependent on substrate stiffness [73], other neuronal types including cortical neurons are insensitive to it [74]. These results suggest that regulation of axonal polarity and growth by NMII is likely affected by mechanical properties of the extracellular matrix, which could govern the activation of one particular NMII isoform in response to external forces. In agreement, environmental elasticity activates Rho signaling and NMII activity in human neurons derived from induced pluripotent stem cells (iPSCs) [75]. NMII activity also regulates the differentiation of mesenchymal stem cells to neurons in response to substrate stiffness [76]. Finally, neuronal activity modulates RhoA and axon pathfinding in response to guidance cues [77,78], so one may envisage that neuronal activity influences the activation of NMIIs. This model would work as a feedback mechanism in which neuronal activity would reinforce axonal outgrowth, thereby increasing and fine-tuning neural connections.

### 3.2. Non-Muscle Myosin II in Neuronal Migration

Migration of newly born neurons from the generation place to the final destination is a critical event during brain development. This process is mediated by guidance factors that regulate intracellular contractile forces driving movement. Neuronal migration involves movement of centrosomes and soma leading the translocation of the nucleus by forces generated by actin, microtubules, and motor proteins [9]. During cell motility, NMII acts in concert with integrin adhesions to provide traction forces to the lamellipodial actin filaments for protrusion and migration [4,79]. In astrocytes, NMII acts downstream of β1 integrin to mediate centrosome integrity during polarization and migration towards the wound after brain damage [80].

In migrating neurons, F-actin and active phosphorylated NMIIB (Ser 19) are enriched in the neuronal leading process surrounding the centrosome and the trailing process in the direction of the migration, where they coordinate the forward movement of the centrosome and soma [33,34,35] (Figure 2). Pharmacological inhibition of NMII motor activity blocks F-actin dynamics and disrupts the forward movement of cells, thereby suggesting that NMII is essential for mediating actin dynamics and providing pulling forces to translocate the soma during neuronal migration [34,35]. This mechanism may explain the neuronal migration defects observed in mutant mice harboring point mutations in the motor domain of NMIIB [50]. Similarly, actomyosin contractile forces mediate nuclear translocation (i.e., nucleokinesis) of interneurons by exerting pulling and pushing forces in the leading and rear cell regions, respectively [81,82] (Figure 2). Furthermore, NMII accumulates at the rear of the soma where it assists dynein to push the nucleus forward along the trailing microtubules in migrating neural precursors [83,84]. The physiological role of the myosin motor activity mediating the forward movement of the nucleus is further supported by the effect of blebbistatin and actin-depolarizing compounds to block nuclear translocation in migrating neurons [81,83]. Besides translocation, the nucleus also rotates through the association of cytosolic dynein and kinesin-1 with nuclear nesprins, as shown in cerebellar granule cells [84]. Nuclear rotation towards the direction of translocation is generated by microtubules through the linker of nucleoskeleton and cytoskeleton (LINC) complex [84]. Whereas LINC complex can also bind to actin, blocking actin contractility with blebbistatin drastically reduces the speed and angle motion of the nucleus during rotation in fibroblasts [85]. Thus, it is conceivable that some parameters of nucleus rotation may be regulated by NMIIs in neurons.

Actomyosin also governs nuclear cytoskeleton and organization [86,87]. The actin depolymerizing factors cofilin-1 and ADF, which induce F-actin disassembly, maintain nuclear morphology and integrity through the LINC complex. This complex associates with the inner and outer membrane of the nucleus, working as a bridge between the cytoskeleton and the nucleoskeleton. This regulation depends on ROCK-mediated NMII activity rather than F-actin organization [88,89]. Among other mechanical elements, the cell also requires NMII for nuclear shape and chromatin organization [90]. Several studies indicate that NMII regulates gene expression and/or transcriptional complexes in a variety of cell types, especially involved in oncogenesis [91,92,93]. Whether similar regulation of gene expression occurs in neurons or other brain cell types is unclear.

### 3.3. The Synaptic Function of Non-Muscle Myosin II

#### 3.3.1. The Role of F-Actin in Synapse Morphology

The capability of neurons to process, store, and retrieve information depends critically on functional and morphological changes of synapses. The formation, stability, and structural remodeling of synapses is controlled by rearrangements of neuronal cytoskeletal elements, including actin filaments, microtubules, neurofilaments, and scaffold proteins (reviewed in [94]). Actin and actin-associated and actin-regulatory proteins are the main structural components of excitatory synapses and to a minor extent of inhibitory shaft synapses. Assembly and polymerization of F-actin filaments is essential for formation, maturation, and function of synapses, especially at dendritic spines, the main sites of postsynaptic excitatory glutamatergic neurotransmission and plasticity [95,96,97].

In glutamatergic synapses, local activation of postsynaptic N-methyl-D-aspartate receptors (NMDAR) and Ca^2+^ influx mediates activation of Ca^2+^/calmodulin-dependent protein kinase II (CaMKII), which in turn phosphorylates small Rho GTPases and modulates F-actin polymerization [98]. Activation of Rac1 and Cdc42 stimulates F-actin polymerization, leading to spine formation and enhancement, whereas RhoA activation induces spine loss by activating ROCK and actomyosin reorganization [98] (Figure 3). It is still unclear, however, how distinct Rho GTPases act coordinately to maintain and reorganize actin dynamics during structural synaptic plasticity. Synaptic activity also regulates actin dynamics and this is crucial for synapse plasticity in the hippocampus [99,100]. Accordingly, activity-dependent synaptic plasticity induces changes in F-actin dynamics in dendritic spines [101]. Real-time single synapse resolution imaging was recently employed to visualize the reorganization of cytoskeletal proteins at synapses that occurs during enlargement of spine volume in neurons of the hippocampus after long-term potentiation (LTP) [102]. In an initial phase, F-actin and actin-modifying factors (cofilin-1, Aip1, actin-related protein 2/3—Arp2/3) rapidly increase at spines, whereas some F-actin-stabilizing factors (profilin, debrin, GluA1, α-actinin, and CamKII) decrease. Then, LTP affects the content of F-actin branching, through the Arp2/3 complex, or capping ABP1 proteins, which allows a switch in the equilibrium from actin stabilizers to actin modifiers during synaptic plasticity, increasing its susceptibility to reorganization. Altogether, these results indicate that the ability of actin to polymerize and depolymerize provides an efficient way for coupling synaptic activity to plastic structural changes at synapses.

#### 3.3.2. NMII in Synapse Formation and Function

**Figure 4:**
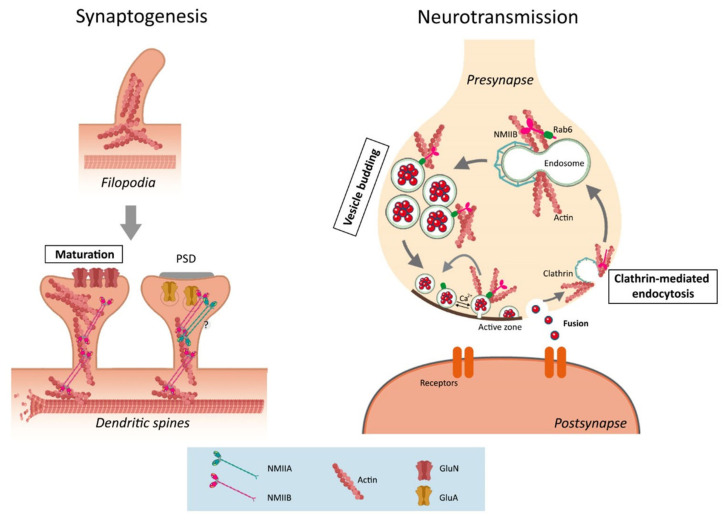
NMII regulates synapse formation and neurotransmission. Left: Incorporation of NMIIB at dendritic filopodia mediates spine stability and NMDAR/GluN-dependent spine maturation. The role of NMIIA in spine morphogenesis is still unknown (indicated with a question mark). PSD: postsynaptic density. Right: NMIIB plays a key role in neurotransmission by participating in multiple presynaptic mechanisms, including synaptic vesicle trafficking; vesicle delivery to the active zone; and clathrin-mediated endocytosis and recycling. Roles of NMII in Rab6-mediated vesicle budding from synaptic endosomes and vesicle retrieval have also been hypothesized but not yet demonstrated [109].

Synapses contain actin structures and cytoskeletal motor proteins, including high levels of NMIIB at pre-synaptic and post-synaptic compartments [36,103,104,105,106,107]. The dynamic properties of actin and actin-binding proteins point to a role of these cytoskeletal molecules in synapse formation, maturation, and/or plasticity. The presence of active phosphorylated RLC (Ser 19) at dendritic spines and the increase of synapses and postsynaptic/presynaptic clusters by an active RLC mutant in developing hippocampal neurons suggest that NMII actively participates in synaptogenesis (Figure 4). Indeed, blebbistatin or NMIIB ablation critically affects synapse number and morphology in hippocampal neurons [108,109]. However, what is the specific role of NMII in synaptogenesis and how does actomyosin contractility regulate synapse formation and/or maturation?

During synapse formation, dendritic filopodia are transformed to mature spines through shortening of filopodia and swelling of the filopodia tip, which drives head expansion [110]. It is well established that the increase of stable synaptic pool of F-actin correlates with the size of dendritic spines during maturation of hippocampal neurons [111]. This process requires branching of actin filaments distributed along the dendritic filopodia through the Arp2/3 complex, capping protein, and NMII. This cytoskeletal network complex mediates transformation of dendritic filopodia to mature spines [37]. Particularly, swelling of the filopodia tip and head expansion occur via Arp2/3-complex-dependent actin filament branching, whereas filopodia shortening involves NMII [36]. This morphological transition is accompanied by increased NMIIB or changes in its composition at spines and requires actomyosin contractility (Figure 4). Thus, dendritic filopodia contain NMII single molecules and bipolar filaments, whereas the main NMII species present in the bases, necks, and heads of spines are bipolar filaments [37]. Spine maturation involves a dual function of NMII: (i) stabilization of the F-actin pool at the base of the spine head, a process that is independent of contractility, and (ii) contraction/disassembly of non-aligned actin filaments at the spine head’s surface [112]. This dual function of NMII at the spine base and head leads to stabilization of spines and fine-tuning of spine shape and dynamics. Not only does the maturation of excitatory synapses require activity-dependent mechanisms, but also this transformation induced by activity depends on active NMII bipolar filaments [36]. In summary, actomyosin-mediated cytoskeletal changes play a key role in synapse formation and maturation of glutamatergic neurons. However, the relevance of these findings in vivo is still largely lacking, and it will require studies in NMII mutant murine models. Importantly, whether actomyosin regulates synaptogenesis in other excitatory or in inhibitory neurons is unknown.

What are the specific functions of NMIIs at pre-synapses and post-synapses? At pre-synapses, NMII facilitates the delivery of synaptic vesicles to active zones in the neuromuscular junctions of mammals and invertebrates [113,114]. In peripheral synapses, NMII enhances the evoked response under low-frequency stimulation and mediates synaptic vesicle motility and synaptic transmission under high-frequency stimulation. The fact that genetic or pharmacological NMII inactivation decreases evoked responses in the *Drosophila* neuromuscular junction points to a crucial role NMII in neurotransmission [114]. In sympathetic neurons, synaptic transmission depends on presynaptic NMIIB, as revealed by the attenuating effects of blocking presynaptic NMIIB on acetylcholine release [115]. At central glutamatergic synapses, presynaptic NMIIB does regulate spontaneous neurotransmitter release but it is required for neurotransmitter release induced by action potentials [109] (Figure 4). NMIIB regulates neurotransmission by facilitating synaptic vesicle motility and release, as suggested by disruption of directionality and speed of vesicle trafficking by blebbistatin and MLCK inhibitors [116]. Alternatively, NMII can mediate neurotransmission by regulating vesicle retrieval and recycling. Super-resolution and transient motion imaging demonstrate that NMIIB supports synaptic transmission by promoting vesicle endocytosis during sustained neuronal activity in hippocampal synapses [116]. Indeed, synaptic vesicle endocytosis requires the presence and motor function of NMII at the presynaptic compartment. Presynaptic NMIIB promotes retrieval of synaptic vesicles through clathrin-mediated endocytosis, a mechanism that maintains the vesicle recycling pool and synaptic strength in hippocampal synapses [109]. In agreement, genetic or pharmacological inactivation of NMIIB at the presynaptic region disrupts vesicle retrieval in response to stimulation. While these results suggest that NMII motor function participates in vesicle retrieval, the role of NMII in other types of synaptic vesicle endocytosis (e.g., kiss-and-run) and the mechanisms involved are unknown. It is possible that NMII may regulate synaptic vesicle docking, fusion, and retrieval by binding or regulating key synaptic proteins of the SNARE complex, and clathrin or dynamin, respectively. It is interesting to note that binding of NMII to the Rab6 GTPase positively regulates vesicle fission and trafficking on the Golgi network [117]. Therefore, it is possible that Rab6 could mediate NMII-dependent vesicle retrieval from the plasma membrane through a kiss-and-run pathway and vesicle budding from synaptic endosomes, as recently proposed [109]. Altogether, this evidence points towards an essential role for NMII in regulating trafficking, exocytosis, and endocytosis of synaptic vesicles (Figure 4). However, the specific molecular mechanisms by which NMII promotes synaptic vesicle trafficking, fusion, and retrieval remain unclear.

In the postsynaptic region, NMIIB regulates the maturation, morphology, and function of dendritic spines. The localizations of NMIIB at dendritic filopodia and thin, stubby, and mushroom spines likely reflect its multi-faceted functions in spine morphogenesis [118]. As described above, NMIIB facilitates the NMDAR-mediated transit of filopodia and immature spines to mature mushroom-like spines [36,118] (Figure 4). Lack of *Myh10* results in decreased stubby spines and increased spine neck length and head protrusions in hippocampal neurons [119]. NMIIB controls dendritic spine maturation by two main mechanisms: (1) crosslinking and contracting actin filaments, and (2) facilitating actin filament translocation to spines. Accordingly, the non-contractile NMIIB R709C mutant leads to persistence of filopodia-like spines and reduces postsynaptic density (PSD) maturation [118]. Furthermore, mice lacking the NMII B2 alternative splice variant, which binds to actin but lacks actin-activated ATPase activity and motility in vitro [120], show mislocalization and reduction of dendritic spines and branches of Purkinje cells, leading to impaired motor coordination [121].

The mechanism of myosin-dependent spine maturation involves NMDAR/ROCK-dependent phosphorylation of RLC at Thr 18 and Ser 19 [122]. Phosphorylations of these two residues promote myosin ATPase activity and contractility, resulting in the formation of actin bundles and mature mushroom spines with enlarged PSD [118], and the expression of a constitutively active phosphorylated RLC mutant increases the number of dendritic spines in differentiated hippocampal neurons [108]. By contrast, ROCK inhibition leads to increased spine length and filopodia-like spines [118]. It seems that myosin’s capability to contract actin filaments is essential for activity-dependent dendritic spine maturation of hippocampal synapses. In agreement, inhibition of NMII activity or a phosphorylated-deficient RLC mutant prevents spine maturation in the presence of NMDAR activation [118]. Interestingly, NMDAR-induced drebrin/F-actin exodus triggers actin polymerization and dendritic spine enlargement through NMII-dependent remodeling of potentiated spines [123]. Besides its role in synapse remodeling, NMII also modulates spine morphogenesis by acting downstream of the Rho GTPase Rac. Rac regulates positively dendritic spines by phosphorylating and activating NMII via a signaling complex formed by PAK, G protein-coupled receptor kinase interacting ArfGAP 1 (GIT1), and PAK interacting exchange factor (PIX) [108]. Inhibition of PAK or NMII decreases the number of mature spines and synapses [36,108,118]. How these distinct regulatory pathways are integrated to mediate structural and functional changes of dendritic spines is still an important unresolved question.

In mature hippocampal synapses, NMIIB controls spine dynamics by increasing the rates of spine expansion and retraction. Pharmacological or genetic NMIIB inactivation lead to transformation of mushroom-like spines to filopodia, a phenomenon that is accompanied by reduced synaptic AMPA puncta and impaired alpha-amino-3-hydroxy-5-methyl-4-isoxazole propionic acid receptor (AMPAR)-mediated excitatory synaptic transmission [36]. Therefore, besides its critical role in dendritic spine formation and maturation, NMIIB acts postsynaptically to maintain spine morphology in mature synapses. Whether AMPAR, RhoA/ROCK, or alternative molecular mechanisms mediate NMIIB-dependent spine formation, maturation and stability is still unclear. More intriguing is whether other NMII isoforms act similarly to NMIIB in synapse formation and function.

#### 3.3.3. Roles of NMII in Synaptic Plasticity and Memory

NMII has emerged as an important regulator of synaptic strength since it mediates changes in the molecular components, structure, and function of synapses. This is evident in experiments of cultured hippocampal neurons and brain slices that show decreases in the frequency and amplitude of miniature excitatory postsynaptic currents (mEPSCs) after genetic or pharmacological *Myh10* inactivation [36,119]. Genetic ablation of *Myh10*, but not *Myh9*, also impairs excitatory synaptic transmission, presumably by affecting dendritic morphology during maturation of hippocampal neuronal circuits [124]. NMIIB regulates synaptic transmission and plasticity at hippocampal synapses, acting likely through pre and post-synaptic mechanisms [36,109]. However, it is unclear whether NMII regulates different forms of synaptic plasticity in other neuronal circuits. Therefore, we next describe the molecular mechanisms by which NMII regulates synaptic plasticity in the hippocampus.

NMIIB is required at postsynaptic regions to maintain NMDAR-dependent synaptic plasticity in the hippocampus. Thus, shRNA viral-mediated *Myh10* inactivation impairs the early phase of LTP but not synaptic transmission in hippocampal slices by reducing F-actin accumulation at spines [122]. Mechanistically, myosin II ATPase activity contributes directly to the dynamic turnover of stable filaments at synapses. This establishes an equilibrium between stable and unstable filaments at dendritic spines that allows for stability of synaptic plasticity. Therefore, NMIIB can promote actin-dependent spine remodeling following LTP, a process necessary for stabilization of synaptic plasticity. The molecular mechanisms underlying NMIIB-mediated regulation of synaptic plasticity involve myosin activation by RLC phosphorylation that occurs after NMDA and LTP stimulation [122]. Notably, myosin RLC binds to the C-terminal of GluN1 and GluN2 subunits of NMDARs, an interaction displaced by Ca^2+^/calmodulin [125]. Although the physiological relevance of this interaction is still unclear, it may contribute to the motility and trafficking of NMDAR during LTP [126]. NMIIB and MLCK-mediated regulation of actin dynamics is essential for the synaptic incorporation of NMDAR during synaptic plasticity [127]. By contrast, the binding of filamin A-interacting protein (FILIP) near the NMIIB ATPase domain interferes with its actin-binding activity and synaptic localization, leading to decreased synaptic GluN1 and GluN2A [128]. Similarly, inhibition of NMII ATPase activity suppresses NMDAR synaptic incorporation and EPSCs during PKC and theta burst-induced synaptic plasticity [127]. At present, it is not completely clear whether regulation and trafficking of postsynaptic glutamate receptors is mediated by structural and/or contractile NMII functions. However, it is possible that NMII contributes to the maintenance of synaptic plasticity by binding and directly regulating postsynaptic NMDARs.

In agreement with the established concept that synaptic plasticity is a cellular correlate of memory, one can envisage that NMIIB is required for both hippocampal LTP and memory. While NMIIB inactivation impairs hippocampal synaptic plasticity (e.g., LTP) and consolidation, but not acquisition of contextual fear-associated memories, pretreatment with the actin polymerization activator jasplakinolide before training prevented blebbistatin-induced disruption of long-term contextual memory and LTP [122]. Although this result strongly supports the idea that NMIIB contributes to memory consolidation by facilitating actin dynamics, there are some apparent discrepancies about the role of myosins in fear memories. Some studies indicate that NMIIs are essential only for consolidation of long-term fear memories in the lateral nucleus of the amygdala [129], whereas other authors suggest a role for MLCK in preventing acquisitions of fear memories [130]. Besides, pharmacological MLCK inhibition in the lateral amygdala before conditioning enhances both short-term and long-term fear memory. This effect is accompanied by increased LTP in the amygdala, and it is not observed if MLCK inhibition occurs after conditioning, which suggests that MLCK prevents fear memory formation [130]. Whether MLCK regulates synaptic plasticity and memory through myosin or other downstream effectors remains to be established. NMIIB contributes to the maintenance of drug abuse-associated memories in the basolateral amygdala by mediating synaptic actin polymerization [131,132]. Accordingly, blebbistatin and genetic *Myh10* inactivation disrupt methamphetamine-associated memory during context-induced drug seeking, an effect that is not observed in auditory fear memory [133]. The facilitating effect of NMII on drug abuse-induced memory is associated with an increase of dendritic spines and is dependent on the drug type [134]. Therefore, the role of NMII in processing some specific types of memory (fear memory, drug abuse-related memory, etc.) involves regulation of synaptic plasticity. Together, these studies suggest that NMIIB is a critical regulator of actin filament dynamics during synapse remodeling and plasticity, and memory.

## 4. Non-Muscle Myosin II in Neurological and Neurodegenerative Diseases

### 4.1. NMIIs in Neurodevelopmental Diseases

The essential functions of NMII and its effectors in cytoskeletal dynamics point towards a pathological role of myosin in multiple human diseases. *MYH9*, *MYH10,* and *MYH14* mutations are reported in a variety of human diseases ([4], for review). Particularly, *MYH9* mutant carriers develop deafness, nephritis, and macrothrombocytopenia [135]. Several *MYH14* mutations in the head and tail domains have been similarly reported in autosomal-dominant deafness cases [136]. These mutations show a general reduction of Mg^2+^-ATPase activity and a disruption of actin filament translocation, suggesting that motor dysfunction may underlie these pathologies. NMII and their effectors were recently associated with several brain disorders, including neurodevelopmental, neurodegenerative, and brain injury diseases (Table 2).

Genetic analyses have revealed de novo *MYH9* mutations associated with neuropsychiatric disorders [138,139]. *MYH9* is one of the three genes together with *LRP1* and *POGZ* shared by autism, schizophrenia, and intellectual disability [139]. How these mutations cause these distinct neurological disorders is still unknown, but understanding the downstream effects of these new *MYH9* mutations could shed light on the mechanisms underlying these pathologies. Whole exome sequencing (WES) has also identified a rare mutation in *MYH10* linked to mental retardation and pathological features of dysplasia, microcephaly, cerebral atrophy, and hydrocephalus [142]. This mutation is probably deleterious because it results in premature termination and truncation of NMIIB. A similar phenotype is partially modeled by germline deletion of *Myh9* in mice [44], which suggests that loss of *Myh* function causes severe developmental cerebral defects. More recent proteomic studies have identified NMIIA, B, and C as binding partners of rotatin (*RTTN*)*,* the gene of which is mutated in some cerebral pathologies, such as severe intellectual disability, cortical malformation, microcephaly, and polymicrogyria with seizures [149]. Although the specific mechanisms by which rotatin mutations trigger neurodevelopmental malformations remain unclear, it is tempting to speculate that these defects could be partially mediated by NMII. Despite this genetic evidence, the involvement and specific contributions, if any, of these de novo myosin mutations on neuropsychiatric diseases need further confirmation. More importantly, the underlying cellular mechanisms by which disruption of NMII underlies neurological diseases remain to be identified.

Synapse pathology, characterized by synapse dysfunction, morphological changes, or loss, is a common pathological feature of several neurodevelopmental and neurodegenerative diseases [151]. Considering that NMIIB is required for formation and maturation of dendritic spines, one can envisage that NMII dysfunction may lead to synapse dysfunction and/or loss in neuropsychiatric disorders. In this context, it is relevant that *PAK3*, which phosphorylates and activates RLC, is mutated in cases of X-linked mental retardation [152,153,154]. *PAK3* is a critical regulator of synapse formation and plasticity in the hippocampus, whereas a mental retardation-linked *PAK3* mutant causes elongated dendritic spines and reduced mature synapses, spontaneous EPSCs, and reduced AMPAR levels [108,155]. Rac acts through the GIT1/αPIX/PAK complex to mediate NMII activation during spine and synapse formation [108]. The specific mechanisms underlying synapse pathology caused by mutant *PAK3* and the possible involvement of NMII are unknown. A plausible interpretation of the above results is that synaptic defects caused by PAK3 mutations through NMII might contribute to cognitive deficits in some mental retardation cases. In support of this idea, mutations in the αPIX gene (*ARHGEF6*) also cause X-linked mental retardation [148]. One of these mutations results in a C-terminal truncation of the protein that affects PIX function and PIX/GIT1 synapse mistargeting, which in turn could lead to synapse morphology abnormalities [156]. Indeed, αPIX/*Arhgef6*-deficient mice show loss of synapses accompanied by hippocampal-dependent LTP and memory deficits [157], whereas PAK3 or PIX inactivation results in similar spine morphology changes, suggesting a common mechanism regulating spine morphogenesis [158]. It needs to be confirmed whether these *PAK3* and *PIX* mutants affect synapse formation, stability, and/or morphology, and whether NMII dysfunction participates in the synaptic defects in mental disorders.

Rho GTPases regulate synapse morphology and dendritic arborization, mechanisms that are essential for processing information in neural circuits related to cognition [159]. Rho GTPases have recently emerged as key contributors to synapse and dendritic structural abnormalities in neurodevelopmental diseases, and mutations in its members and associated signaling partners are linked to autism, intellectual disability, and schizophrenia [160,161]. A point mutation in the L-type voltage gated calcium channel Cav1.2 that increases RhoA activity and results in aberrant dendrites causes Timothy syndrome, a neurodevelopmental disease related to autism [137]. This mutation boosts the activity-dependent RhoA-mediated phosphorylation of myosin RLC (Ser 19), thereby enhancing ATPase activity and myosin contraction. The authors nicely showed that a dominant negative RhoA mutant or pharmacological inhibition of RhoA prevents the activity-dependent dendrite retraction caused by the Cav1.2 mutant [137]. The precise role of NMII in Timothy syndrome is not clear, but it is conceivable that mutant Cav1.2 causes neuronal morphology defects through NMII. As detailed above, Rac regulates spine morphogenesis and synapse formation in hippocampal neurons by acting through a GIT1/αPIX/PAK signaling complex that phosphorylates and activates RLC. A recent study also showed that rare *GIT1* variants found in schizophrenia patients cause synaptic defects by disrupting PAK3 activation [162]. Considering that activation of PAK1/3 or NMII rescues the defects in synapses and spine numbers caused by *GIT1* inactivation [108], it is plausible that mutant *GIT1* variants cause synaptic defects by affecting PAK/PIX/GIT1-dependent NMII activation. Alternatively, the reduced number of synapses in schizophrenia could arise from increased myosin activity during spine maturation or refinement. In fact, increased myosin RLC phosphorylation and activity induce dendritic spine abnormalities during critical periods of spine maturation [118], whereas elevated RLC phosphorylation (Ser 19) associated with changes in PAK1 phosphorylation is detected in the anterior cingulate cortexes of schizophrenia patients [147]. A role for PAK in synaptic impairments in mental disorders is further supported by the ameliorating effects of PAK inhibitors in the dendritic spine abnormalities and behavioral deficits in adolescent schizophrenia-like and fragile X-syndrome mouse models [163,164]. Finally, spine morphogenesis and excitatory synaptic transmission require the expression of *oligophrenin-1*, an X-linked mental retardation gene that regulates RhoA/ROCK signaling [165]. ROCK inhibition rescues spine length defects caused by loss of oligophrenin-1, but whether these effects are mediated by myosins is still unclear.

In summary, several signaling pathways related to Rho GTPases are mutated or severely affected in mental retardation, intellectual disability, and schizophrenia. It is possible that dysfunction of these genes may lead to morphological changes and loss of dendritic spines and synapses by disrupting NMII function. It is crucial to decipher the specific contributions of actomyosin cytoskeletal dynamics in the cerebral and behavioral abnormalities occurring in these severe neurological diseases.

### 4.2. NMIIs in Neurodegenerative Diseases

NMII has been also linked to neurodegeneration, particularly to Alzheimer’s disease (AD). AD, the main cause of dementia worldwide, is neuropathologically characterized by the presence of β-amyloid (Aβ) plaques, neurofibrillary tangles of phosphorylated tau protein, and degeneration of axons and synapses [166]. Recent proteomics analyses revealed the interaction of NMIIA and B with tau in CRISPR-Cas9 genome-edited human neuroprogenitor cells [150]. Binding of tau stabilizes the activity and steady-state levels of NMIIB, whereas a frontotemporal dementia-linked mutation decreases NMIIB binding and RLC phosphorylation (Ser 9) [150]. Another interesting link with tau is the finding that the cyclin-dependent kinase-5 (Cdk5) phosphorylates both tau and NMII HC [167]. It was previously shown that myosin VI associates with tau of neurofibrillary tangles in frontotemporal and AD dementias, although the physiological relevance of these interactions is still unclear [168]. This raises the intriguing possibility that pathological phosphorylated tau could affect myosin/NMII structure and/or function, causing pathological neuronal cytoskeletal abnormalities in AD, such as dystrophic neurites, and axonal and synapse degeneration.

It has been recently reported that PS1/γ-secretase, the enzymatic complex responsible for Aβ generation that is mutated in the majority of inherited familial AD cases, promotes axon growth by inhibiting RhoA and promoting NMIIA filament disassembly and cytoskeletal rearrangement in hippocampal neurons [59]. PS1/γ-secretase-deficient neurons show decreased NMIIA phosphorylation (Ser 1943) and NMIIA/actin colocalization in axons, suggesting that loss of PS1/γ-secretase promotes assembly and/or formation of stable actomyosin filaments leading to axon retraction. This may be pathologically relevant because PS1 loss-of-function mutations cause tau-related axonal abnormalities and synapse loss [169], which are early pathological features of both familial and sporadic AD [166,170]. Synapse loss is indeed the best correlate of memory deficits and failure of both pre and post-synaptic mechanisms underlying synapse dysfunction in AD (reviewed in [171]). Interestingly, a recent report using fluorescent deconvolution tomography revealed reduced PAK3 levels at synapses of parietal cortexes of AD and Down syndrome patients [172]. Considering that PAK3 regulates synapse formation and plasticity [155], this result suggests that disruption of synaptic NMII/actin could contribute to early synapse dysfunction and cognitive impairment in these diseases. It is still unclear whether cytoskeletal changes mediated by actomyosin filaments contribute to morphological abnormalities and degeneration of axons, dendrites, and synapses in AD.

### 4.3. NMIIs in Nervous System Injuries

Recent evidence indicates that NMII cytoskeletal changes contribute to neuron death during brain stroke. NMIIA-related actomyosin contractility mediates caspase-3-induced neuronal apoptosis induced by oxidative stress in cerebral ischemia [173]. NMIIA promotes neuronal autophagic death by mediating the interaction of F-actin to autophagy-related gene 9A (ATG9A), a process essential for ATG9A trafficking and autophagosome formation [140]. Alternatively, NMIIA contributes to tight junction morphological changes in endothelial cells during vasculature damage in cerebral ischemia [17,174]. Due to its crucial role in neuronal death and endothelial damage, NMIIA may represent a therapeutic target in cerebral ischemia. In agreement, pharmacological and genetic NMIIA inactivation ameliorates neuronal death, infarct volume, and neurological defects in a mouse model of cerebral ischemia [140,141]. This result strongly suggests that inhibition of actomyosin may be therapeutically efficacious in ameliorating pathological and clinical features associated with brain ischemia.

Several studies indicate a link between NMII and neuron damage and regeneration after neuronal injury. First, ROCK-mediated phosphorylation of RLC in axons around the lesion site is increased after spinal cord injury, which suggests that NMII activation could cause or result in the neuropathology of motoneurons [66]. In support of a causative role, *Myh9* silencing promotes axonal regeneration, synaptic connections, and locomotive function after spinal cord injury in rats [175]. Genetic inactivation of NMIIA and pharmacological NMII inhibition both also promote axon elongation over inhibitory substrates by reorganizing actin and microtubules at the growth cones [28]. In agreement with a beneficial effect of actomyosin disassembly or prevention of its assembly in axon growth, blebbistatin was shown to reverse axon growth defects caused by loss of PS1/γ-secretase in hippocampal neurons [59]. Interestingly, a novel Arg941Leu mutation in the *MYH14* gene in Korean and American families caused peripheral neuropathy leading to muscle weakness and atrophy followed by hearing loss and hoarseness [144,145]. This mutation acts in a dominant-negative fashion to inhibit mitochondrial fission and transport along axons during peripheral neuropathy [146], which suggests that NMIIC is critical for mitochondrial function and dynamics in motoneurons. This mechanism is relevant because impaired mitochondrial function has been tightly linked with peripheral neuropathies and neurodegenerative diseases [176]. Despite this genetic evidence, the involvement and specific contributions, if any, of myosin mutations to central and peripheral axonal neuropathies need further confirmation.

NMII plays a role in axon myelination and remyelination: (i) NMII mediates myelin formation in Schwann cells and sheath coverings of axons in the peripheral nervous system [21]; (ii) NMII affects negatively oligodendrocyte branching and myelination [20,21]; and (iii) genetic ablation of NMIIB in oligodendrocytes promotes axon remyelination and reduces lesion volume after a demyelination injury [143]. For instance, NMII inhibits cell surface extension and spreading of myelin-membrane sheets over non-permissive substrates in oligodendrocytes, and inhibition of actomyosin contractility reduces spreading of myelin-membrane sheets [177]. Pharmacological inhibitors of NMII activating kinases (e.g., ROCK) ameliorate axonal degeneration, autophagy, and synaptic and glial dysfunction in animal models of neurodegeneration [178]. Together, pharmacological modulators of NMII and growth cone cytoskeletal components may be potential therapeutic approaches for promoting axon regeneration and remyelination during neuronal damage and degeneration, such as those occurring in spinal cord injury, amyotrophic lateral sclerosis, and multiple sclerosis.

### 4.4. NMII in Glial Cells and Inflammation

NMIIs play important physiological roles by acting in glial cells during inflammation and injury of the nervous system. Microglia are resident macrophages of the nervous system that are derived from local microglial expansion or differentiation of recruited blood circulating monocytes [179]. In inflammatory and damage conditions, microglia are increased and activated in the brain in a process named reactive microgliosis. NMII activity is critical for cytoskeletal changes in microglia during activation, migration, and phagocytosis in inflammatory and demyelinating conditions [19], suggesting that NMII may play a central role in microgliosis. Interestingly, the interaction of NMIIA/actin with the purinergic receptor P2X7 mediates phagocytosis in human monocytes, whereas the natural P2X7 ligand ATP, and actin polymerization and myosin activity blockers, inhibit P2X7-mediated phagocytosis [180]. This raises the possibility that NMIIA/actin cytoskeletal dynamics may participate in P2X7-mediated phagocytosis in cells of myeloid origin, such as microglia. In support of this idea, innate P2X7-mediated phagocytosis of blood monocytes is decreased in AD, particularly in patients showing more amyloid pathology [181]. Whether NMII contributes to microglial phagocytosis mediated by P2X7 or other mechanisms do, may have important pathological and therapeutic implications in neurodegenerative diseases. Nonetheless, deficits in P2X7-mediated phagocytosis are evident during aging and pathological conditions, such as inflammation and AD [182]. Inhibition of P2X7-mediated proinflammatory responses by P2X7 antagonists or genetic deletion has proven beneficial in several mouse models of neurodegenerative diseases [182]. This has provided a framework for P2X7 targeting as a therapeutic approach in AD [183]. In support of a role of actomyosin on microglial phagocytosis, it has been reported that F-actin accumulates close to the microglia nucleus in engulfing gliapses [184]. Notably, ROCK/Cdc42 signaling mediates phagocytosis of degenerating dopaminergic neurons in a mouse model of Parkinson’s disease, whereas ROCK inhibition prevents it [184]. This result supports the view that inhibition of RhoA/ROCK-mediated actomyosin activation may be beneficial for reducing neuron loss in neurodegenerative diseases [178]. By contrast, a recent study demonstrated increased microglial activation, synapse and neuron loss, and synaptic plasticity and memory deficits in mice with genetic ablation of RhoA in adult microglia [185]. Based on these results, some caution should be taken regarding the use of RhoA inhibitors as a therapeutic approach to ameliorating inflammation and neuron loss in neurodegenerative diseases.

NMII is also critical for blood–brain barrier integrity and permeability by acting in endothelial cells and astrocytes. NMII contributes to PKC-β/RhoA/ROCK-mediated damage of microvasculature during cerebral ischemia [174], and MLCK mediates interleukin-1β-induced blood–brain barrier dysfunction by repressing the expression of the tight junction protein claudin-5 through β-catenin/forkhead box protein 01 (FoxO1) signaling in endothelial cells [186]. Although the specific NMII isoform responsible for regulation of blood–brain barrier is unknown, it is plausible that NMIIA plays a prominent role based on its high levels in cerebral endothelial cells (Figure 1B), and its participation in endothelial tight junctions in brain ischemia [17]. It should be noted that these prior studies were performed mainly in cultured cellular models, so it is critical to confirm the involvement of NMII in blood–brain barrier regulation in relevant in vivo models, including mutant mice or organoids.

On the other hand, astrocytes extend their processes and migrate towards the injured tissue in response to brain damage. Astrocyte polarization and migration towards the wound after brain damage is mediated by β1 integrin via NMII [80]. Accordingly, blebbistatin inhibits integrin-mediated centrosome assembly and polarization in astrocytes. Astrocyte morphological changes mediated by rearrangement of cytoskeletal elements in response to proinflammatory agents involve regulation of actin-interacting elements, including NMII [187]. Future studies aimed at elucidating the biological functions and regulatory mechanisms of NMII isoforms in glial cells are critical for a better understanding of their roles in the physiology and pathology of the central and peripheral nervous systems.

## 5. Concluding Remarks

Multiple cellular mechanisms associated with NMII motors are fundamental for nervous system development, physiology, and pathology. Actomyosin cytoskeletal changes are essential for neuronal development and function, and they also play important functions in glial cells, including inflammation (microglia), repair (astrocyte), and blood–brain barrier integrity (endothelial cells). Further insights into the specific roles of NMII isoforms in the diversity of cell types of the nervous system are key to understanding the full functions of these motor proteins in this tissue. It is intriguing, however, that despite sharing similar functions in actin dynamics, NMII isoforms play opposite functions in neurons as well, including neuronal adhesion and axon and neurite growth, and myelination [21,63]. Distinct roles of NMIIA and NMIIB in spreading and migration of cancer cells have been previously described [188]. These functions may not only rely on the generation of forces that modulate cell shape, but also, they may contribute to the mechanotransduction cascades that activate downstream signaling pathways, which in turn, could affect gene expression. Therefore, besides motor activity, NMIIs may contribute to some neuronal and glial functions by acting directly or indirectly through alternative mechanisms (scaffold proteins, binding partners, gene expression changes, etc.). Generation of novel, cell-type-specific, NMII-mutant genetic mouse models should provide a better understanding of the specific roles of NMIIs in neurons and glial cells in nervous system physiology and pathology.

A large number of neurological disease-linked genes regulate signaling pathways affecting myosin function, whereas *MYH* mutations are associated with intellectual disability, autism, and schizophrenia. NMII also interacts with rotatin, whose mutations cause microcephaly and intellectual disability, and tau, a central protein in AD and frontotemporal dementias [149,150]. The contributions of these mutations to myosin dysfunction in neurological and neuropsychiatric diseases need further confirmation. The central role of NMIIs in the pathogenic mechanisms of neurological and neurodegenerative diseases opens up the possibility for their use as therapeutic targets in these severe brain disorders. For instance, NMIIs play a critical role in axon regeneration and remyelination, and their inhibition protects against axonal degeneration in spinal cord injury [175]. Pharmacological modulators of NMII may be effective targets for promoting axon regeneration and remyelination in nervous system injuries and degeneration, such as amyotrophic lateral sclerosis and multiple sclerosis. Therapeutics based on NMII are challenging though, considering its multiple roles in several tissues, although targeting specific isoforms and/or downstream effectors may allow for modulation of particular biological processes. As a proof-of-concept, pharmacological NMII inhibition attenuates morphological changes of endothelial tight junctions induced by ischemia [17], whereas NMIIA inactivation ameliorates neuronal death, infarct volume, and neurological defects in a mouse model of cerebral ischemia [140,141]. Furthermore, blebbistatin prevents drug abuse-associated memories, suggesting its therapeutic potential for substance abuse disorders [133]. In summary, understanding the molecular mechanisms underlying the involvement of NMIIs in neurodevelopmental and neurodegenerative diseases will provide critical knowledge with which to develop therapeutic treatments targeting actomyosin in nervous system disorders.

## Figures and Tables

**Table 1:** Expression of NMII isoforms in human tissues.

Gene	Protein	Central Nervous System	Other Organs
*MYH9*	NMIIA	Cerebellum	Colon, kidney
		Mesencephalon	lung, platelets
		brain stem	spleen, thymus, uterus
*MYH10*	NMIIB	Cerebral cortex	Aorta
		hippocampus	Kidney
		thalamus, spinal cord	urinary bladder
*MYH14*	NMIIC	Cerebellum	Colon, heart
		corpus callosum	kidney, liver
		pons, thalamus	skeletal muscle

Based on data from references [10,11,12,13].

**Table 2:** Pathological features of neurological diseases associated with NMIIs.

Gene	NMII	Pathological Features	Neurological Diseases	References
*Cav1.2*	NMII	Dendrite retraction	Timothy syndrome	[137]
*MYH9*	NMIIA	Neurological impairments	Autism, intellectual disability, schizophrenia	[138,139]
*MYH9*	NMIIA	Autophagic neuron death, infarct volume, neurological defects	Brain ischemia	[17,140,141]
*MYH10*	NMIIB	Dysplasia, microcephaly, cerebral atrophy, and hydrocephalus	Mental retardation	[142]
*MYH10*	NMIIB	Axon demyelination	Multiple sclerosis *	[21,143]
*MYH14*	NMIIC	Neuropathy, axonal transport deficits, muscle weakness/atrophy	Peripheral neuropathy	[144,145,146]
*PAK1*	NMII	Synapse pathology	Schizophrenia	[147]
*PAK3*/*GIT1/Rac*	NMIIB	Synapse pathology	X-linked mental retardation, schizophrenia	[108]
αPIX/*ARHGEF6*	NMIIB	Synapse pathology	X-linked mental retardation	[108,148]
*PSEN1*	NMIIA	Axon retraction	Alzheimer’s disease *	[59]
*RTTN*	NMIIA,B,C	Cortical malformation, microcephaly and polymicrogyria	Intellectual disability	[149]
Tau	NMIIA,B	Dystrophic neurites and axons	Alzheimer’s disease, FTD *	[150]

* Implication of NMII in these diseases needs further confirmation.
